# A summary of molecular genetic findings in fructose-1,6-bisphosphatase deficiency with a focus on a common long-range deletion and the role of MLPA analysis

**DOI:** 10.1186/s13023-016-0415-1

**Published:** 2016-04-21

**Authors:** René Santer, Marcel du Moulin, Tatevik Shahinyan, Inga Vater, Esther Maier, Ania C. Muntau, Beat Steinmann

**Affiliations:** Department of Pediatrics, University Medical Center Hamburg-Eppendorf, Martinistraße 52, D - 20246 Hamburg, Germany; Arabkir Institute of Child and Adolescent Health, Yerevan, Armenia; Institute of Human Genetics, University of Kiel, Kiel, Germany; Department of Pediatrics, University Children’s Hospital, Munich, Germany; Department of Pediatrics, University of Zurich, Zurich, Switzerland

**Keywords:** Fructose bisphosphatase, *FBP1* gene, MLPA, Turkey, Armenia

## Abstract

**Background:**

Fructose-1,6-bisphosphatase deficiency is a rare inborn error of metabolism affecting gluconeogenesis with only sporadic reports on its molecular genetic basis.

**Results:**

We report our experience with mutation analysis in 14 patients (13 families) with fructose-1,6-bisphosphatase deficiency using conventional Sanger sequencing and multiplex ligation-dependent probe amplification analysis, and we provide a mutation update for the fructose bisphosphatase-1 gene (*FBP1*). Mutations were found on both chromosomes in all of our 14 patients including 5 novel mutations. Among the novel mutations is a 5412-bp deletion (c.-24-26_170 + 5192del) including the entire coding sequence of exon 2 of *FBP1* that was repeatedly found in patients from Turkey and Armenia which may explain earlier poorly defined findings in patients from this area. This deletion can be detected with specific primers by generation of a junction fragment and by MLPA and SNP array assays. MLPA analysis was able to detect copy number variations in two further patients, one heterozygous for a deletion within exon 8, another heterozygous for a novel deletion of the entire *FBP1* gene.

**Conclusions:**

Based on our update for the *FBP1* gene, currently listing 35 mutations worldwide, and knowledge of PCR conditions that allow simple detection of a common *FBP1* deletion in the Armenian and Turkish population, molecular genetic diagnosis has become easier in FBP1 deficiency. Furthermore, MLPA analysis may plays a useful role in patients with this disorder.

**Electronic supplementary material:**

The online version of this article (doi:10.1186/s13023-016-0415-1) contains supplementary material, which is available to authorized users.

## Background

Fructose-1,6-bisphosphatase (FBP1) deficiency [OMIM: 229700], first described in 1970 [1], is an inborn error of gluconeogenesis. Patients present with ketotic hypoglycemia and lactic acidosis triggered by catabolic episodes such as prolonged fasting and/or febrile infections [2]. Laboratory findings may include hyperalaninemia, hyperketonemia, an increased lactate/pyruvate ratio, an elevated plasma concentration of uric acid, glyceroluria [2], and pseudo-hypertriglyceridemia [3]. FBP1 deficiency is generally believed to be very rare with an estimated incidence between 1 : 350,000 and <1 : 900,000 in the Dutch and French population, respectively [4, 5]; but it may be more frequent in populations with a higher rate of consanguinity.

FBP1 deficiency is inherited in an autosomal recessive way. It is caused by mutations within the *FBP1* gene (OMIM: 611570) which spans approx. 31 kb on chromosome 9q22.2-q22.3 and consists of 8 exons [6]. To date, only a small number of mutations has been published and among them, c.959dupG has been reported to be the most common one in Caucasians but also in patients from Japan and China [5, 7–9].

We report results of mutation analysis of our laboratory, describe how we have characterized a common exon 2 deletion detected in patients with Turkish or Armenian ethnic background, and provide PCR conditions for verification of this deletion which is otherwise not detectable by standard sequencing techniques. Finally, we show for the first time that MLPA analysis may play a useful role in the diagnosis of FBP1 deficiency.

## Methods

Fourteen patients with FBP1 deficiency from 13 families with typical clinical and laboratory results were diagnosed in our laboratory between 2006 and 2014 (Table 1). Not all of them had enzymatic studies performed but all parents gave their informed consent to search for the molecular basis of the disease of their children and to be investigated for their own carrier status. In all of them, all 8 exons and adjacent intronic segments of the *FBP1* gene were amplified by PCR and sequenced according to standard Sanger techniques (primer sequences and PCR conditions available upon request). In those patients in whom we assumed a deletion of exon 2 (the first coding exon), we were able to generate a junction fragment with primers 5′-taaaggtttccgcgattcac-3′ (sn) and 5′-gaccatcctggccaacac-3′ (asn). Results of sequencing studies were compared to our *FBP1* reference sequence NM_001127628.1. Nomenclature for the description of sequence variants follows the recommendations of the Human Genome Variation Society [10]. The bioinformatic tools Polyphen-2 [11] and Mutation Taster [12] were used to predict effects of sequence aberrations.

**Table 1:** Ethnic origin and molecular genetic findings of the 14 patients of this study

Patient number		SANGER sequencing results			MLPA results	
	Ethnic origin	Mutation	Mutation effect		Deletion of …	Reference for first report
1	Armenia^a^	**c.-24–26_170 + 5192del** ^b^	**p.0?**	homo	exon 2 **(homo)**	**This study**
2	Turkey^a^	**c.-24–26_170 + 5192del** ^b^	**p.0?**	homo	exon 2 **(homo)**	**This study**
3	Turkey^a^	**c.-24–26_170 + 5192del** ^b^	**p.0?**	homo	exon 2 **(homo)**	**This study**
4	Pakistan	c.841G > A	p.(E281K)^c^	homo	*n.a.*	[3]
5	Pakistan	c.841G > A	p.(E281K)^c^	homo	*n.a.*	[3]
6	Pakistan	c.881G > A	p.(G294E)	homo	*n.a.*	[18]
7	Pakistan	c.841G > A	p.(E281K)^c^	homo	*n.a.*	[3]
8-1	Germany	c.490G > A	p.G164S	homo	*n.a.*	[8]
8-2	Germany	c.490G > A	p.G164S	homo	*n.a.*	[8]
9	Germany	c.704dupC	p.(D236Rfs*2)	homo	*n.a.*	[7]
10	Turkey /	**c.359C > T**	**p.(P120L)**	hetero	*n.a.*	**This study**
	Turkey	c.881G > A	p.(G294E)	hetero	*n.a.*	[4]
11	Turkey	c.841G > A	p.(E281K)^d^	homo	*n.a.*	[4]
12	Germany /	**c.619G > C**	**p.(G207R)**	hetero	-	**This study**
	Germany	*n.d.*	?	?	exon 8 **(hetero)**	**This study**
13	Germany /	c.959dupG	p.(S321Ifs*13)	hetero	-	[14]
	Germany	deletion^e^	?	hetero	exons 1–8 **(hetero)**	**This study**

^a^see Additional file 1: Fig. S1

^b^only detectable when sequencing a junction fragment with specific primers

^c,d^represents different haplotypes

^e^long range deletion (larger than exon 08) suggested by haplotype analysis

*n.a.*, not applied

*n.d.*, not detected

Novel mutations are shown in bold

In those patients in whom the diagnosis of FBP1 deficiency was not confirmed by Sanger sequencing and the detection of 2 biallelic mutations within *FBP1*, MLPA analysis was performed. We used the reaction mixtures SALSA MLPA probemix P255-B1 ALDOB-FBP1 (MRC Holland, Amsterdam, The Netherlands) according to the manufacturer’s recommendations. Acquired data were normalized with 3–5 control DNA samples isolated in our laboratory. Calculations were performed with the SeqPilot software for genetic analyses version 4.1.2 (JSI Medical Systems, Ettenheim, Germany). SNP array analysis was performed using the Genome-Wide Human SNP Array 6.0 (Affymetrix, Santa Clara, CA, USA) evaluated by the Genotyping Console software version 4.1.

## Results and discussion

Conventional Sanger sequencing analysis of all coding exons allowed the diagnosis of FBP1 deficiency in 9 out of the 14 patients (patients 4–11 in Table 1). These patients were found to be homozygous or compound heterozygous for mutations within *FBP1.* Among them, we found two novel missense mutations, p.(Pro120Leu) and p.(Gly207Arg) in exons 4 and 6, respectively, each in single families. Each of these two amino acid positions are part of highly conserved stretches of amino acids. Polyphen-2 predicts both of these 2 missense mutations to be ‘probably damaging’ (score 1.00). Mutation Taster classifies them as ‘disease-causing’ (with probability scores of 0.99999999999648 and 0.999999999878082, resp.). To our knowledge, p.(Pro120Leu) has never been reported to databases before; according to the ExAC database, the p.(Gly207Arg) variant has been observed in 10 European (non-Finnish) individuals in the heterozygous state with an allele frequency of 0.0001498 [13].

To date, only a limited number of *FBP1* mutations has been detected worldwide; our study brings up the total number to 35 (Table 2). Only few mutations have been found that do not have the characteristics of a private mutation. Among them is c.959dupG, originally found in the Japanese population [14] that has also been detected in patients from Europe [5] and North America [7], and recently also in patients from China [9]. Another example is c.841G > A which has been detected in several unrelated patients from Pakistan [3] but also, with a different haplotype, in patients from Turkey [this study]. Furthermore, c.685C > T has repeatedly been found in seemingly unrelated families from Morocco [5, 15].

**Table 2:** Summary of the 35 *FBP1* mutations reported in fructose-1,6-bisphosphatase deficiency

	Nucleotide change	Amino acid change	Ethnic origin	Reference^a^
Single nucleotide changes				
Exon 2	c.88G > T	p.(E30*)	Japan	[8]
Exon 4	c.359C > T	p.(P120L)	Turkey	**This study**
Exon 5	c.472C > T	p.(R158W)	France	[5]
	c.490G > A	p.G164S	Japan/South Korea/?	[5, 8, 19]
	c.530C > A	p.A177D	Japan	[8]
Exon 6	c.581 T > C	p.(F194S)	Japan	[20]
	c.619G > C	p.(G207R)	Germany	**This study**
	c.639C > G	p.(N213K)	?	[5, 7]
	c.648C > G	p.(Y216*)	Sweden	[18]
	c.685C > T	p.(Q229*)	Morocco	[5, 15]
Exon 7	c.778G > A	p.G260R	Pakistan/Sweden	[18, 21]
Exon 8	c.841G > A	p.(E281K)	Pakistan^b^/Turkey^b^	[3]
	c.841G > T	p.(E281*)	Saudi Arabia	[22]
	c.851C > G	p.(P284R)	Japan	[20]
	c.881G > T	p.(G294V)		[7]
	c.881G > A	p.(G294E)	Sweden/Pakistan	[18/3]
Deletions				
Complete Deletion of the *FBP1* gene^c^		p.0?	Sweden	[18]
Complete Deletion of the *FBP1* gene^d^		p.0?	Germany	**This study**
Exon 2	**c.-24–26_170 + 5192del**	p.?	**Turkey/Armenia**	**This study**
	c.35delA	p.N12Tfs*2	Turkey/Germany (?)	[21]
	c.48delC	p.(F17Sfs*15)	France	[5]
Exon 3–7	complete deletion	p.?	?	[5]
Exon 6	c.616_619delAAAG	p.(K206V*70)	Turkey	[23]
	c.660delT	p.(F220Lfs*57)	Turkey	[24]
Exon 7	c.807delG	p.(K270Rfs*7)	?	[7]
Exon 8	deletion^e^	p.?	Germany	**This study**
	c.838delT	p.Y280Tfs*25	South Korea	[19]
	c.966delC	p.D323Tfs*7	Iran	[21]
Insertions/Duplication				
Exon 2	c.114_119dupCTGCAC	p.(C39_T40dup)	Saudi Arabia	[22]
Exon 6	c.704dupC	p.(D236Rfs*2)	?	[7]
Exon 8	c.865dupA	p.(M289Nfs*45)	Greece	[5]
	c.959dupG^f^	p.S321Ifs*13	Japan/Europe/China	[5, 7–9]
Indel				
Exon 7	c.731_738delins20	p.(R244_Y245delins6)	Turkey	[5]
Splicing				
Intron 4	c.427–1del	p.(K143_P189del)	?	[5]
Intron 7	c.825 + 1G > A	p.?	?	[5]

^a^slash (/) refers to slash in column ‘ethnic origin’

^b^with different haplotypes

^c^together with deletion of *FBP2* and parts of *ONPEP* (hg19 chr9:g.(97295486_97300076)_(97571249_97571455), approx. 0.28 Mb)

^d^together with deletion of *FBP2* (hg19 chr9:g.(97281072_97289359)_(97419146_97420857), approx. 0.13 Mb)

^e^exon 8 only according to additional SNP array analysis (hg19 chr9:g.(97364379_97365560)_(97365642_97365985))

^f^originally named c.960_1insG

Novel mutations are shown in bold

**Fig. 1:**
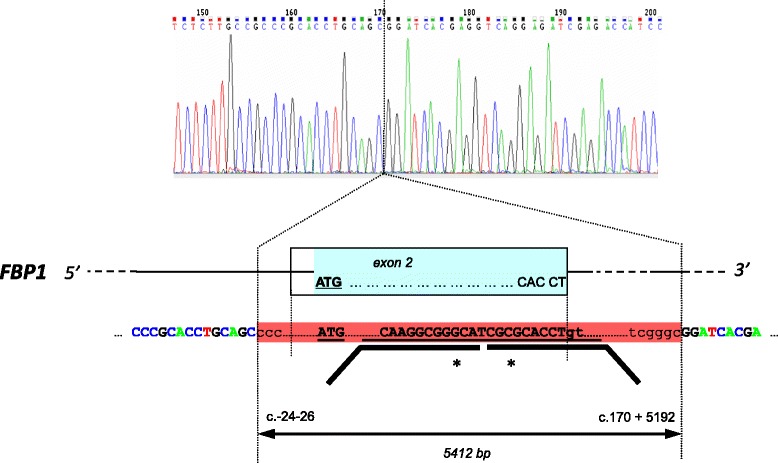
Characterization of a common long-range deletion of the *FBP1* gene. A junction fragment including a deletion in the range of exon 2 was generated from DNA of patient 1. The result of the sequencing reaction is shown. The novel deletion (*indicated in red*) comprises 26 bp of intron 1, another 24 bp of the untranslated region (5′-UTR) before the ATG initiation codon of exon 2, the entire coding region of 170 bp of exon 2 (*blue*), and another 5192 bp of intron 2. The bold black lines (*indicated by the asterisks*) describe the position of the MLPA probes for exon 2 used in this study

In two of our patients, #12 and #13, only one mutation was detected by conventional Sanger sequencing analysis, however, haplotype analysis in the parents of patient #13 already suggested a long range deletion of the paternal allele (*detailed results not shown*). Of note, in 3 consecutive unrelated patients, one from Armenia and two from Turkey, no PCR product could be generated for exon 2 of the *FBP1* gene. This observation prompted us to further investigate these patients. This was of particular interest since earlier reports on mutations in *FBP1* had speculated that deletions within exon 2 (which at that time was termed exon 1) are common in the Turkish population, although the authors were not able to further characterize them [7]. Since we assumed the presence of a long-range deletion in these 3 patients, extensive modification of primer pairs was performed with the aim to generate a PCR product of acceptable size to be visible on polyacrylamide gel electrophoresis and eventually allowed the successful generation of a junction fragment (Fig. 1). All 3 patients in whom exon 2 could not be amplified with standard primers were thus found to be homozygous for a large deletion spanning 5412 base pairs and including the entire coding sequence of exon 2 (c.-24-26_170 + 5192del). All these patients were seemingly homozygous for the following polymorphisms that are all known from databases and have also been detected in our lab both in healthy and diseased controls: c.426 + 7T [rs8192689], c.567 + 31G [rs3739747], c.651T [p.(=), rs1042144], c.653A [p.(Arg218Lys), rs1769259], c.705 + 14C [rs2297084], c.960G [p.(=), rs1769257], c.*213T [rs9695]. Segregation analysis showed that all the patients’ parents carried the deletion in the heterozygous state and indicated that a single haplotype was associated with this deletion (Additional file 2: Fig. S3). These results are compatible with our assumption that this mutation represents a founder mutation in the Armenian and Turkish population. We believe that this mutation plays quite an important role in that geographical area since, in addition to Herzog et al. [7] (*see above*) who supposed deletions in exon 2 in patients originating from Turkey, also Lebigot et al. [5], in a most recent study, reported exon 2 deletions by gene dose assays in Turkish patients; again, no further details regarding its length and location were provided. Furthermore, a preliminary communication from Turkey reported a relatively high number of FBP1-deficient cases from this region and, again, mentioned poorly defined exon 2 deletions [16]. It may therefore be speculated that the deletion characterized in detail in this paper is the same deletion as originally mentioned by several authors [5, 7, 16] and it may be concluded that this deletion of exon 2 is a relatively common cause of FBP1 deficiency in patients of Turkish and Armenian origin. Patients with this ethnic background should primarily be screened for this deletion and Sanger sequencing is now possible when using specific primers that allow sequencing of a junction fragment.

**Fig. 2:**
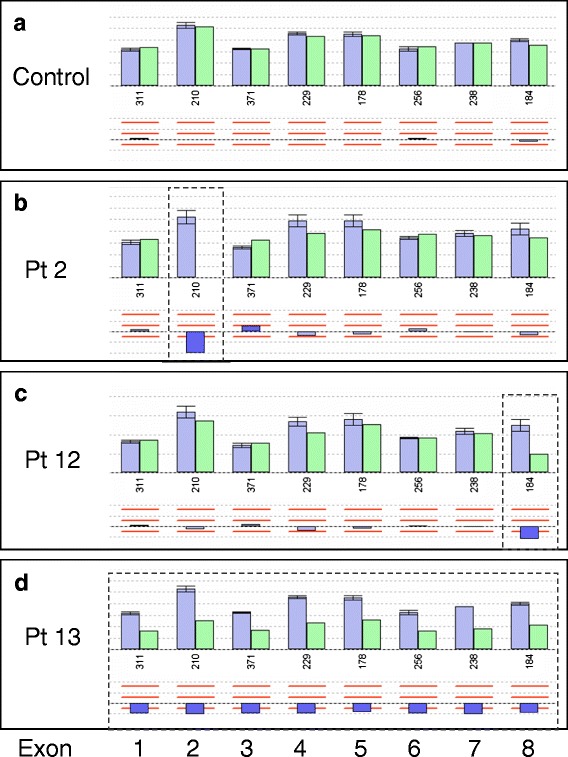
Results of MLPA analysis in FBP1 deficiency. Results are shown for a control sample (**a**), patient 2 (**b**), patient 12 (**c**), and patient 13 (**d**). For patient details *see* Table 1. Each panel shows the results for the intensity of probe amplification for the eight exons of *FBP1*. Patients’ results are depicted in green bars, while means (± SD) of concomitantly measured controls are shown in blue. The presentation below these bars shows the deviation of patients’ results as a percentage of control with the dotted line representing 0, and the horizontal red bars −25 %, +25 %, and +50 %, respectively. *Note* the missing probe amplification for exon 2 in patient 2 which is in line with homozygosity for the novel exon 2 deletion. Patient 12 shows a signal intensity for exon 8 of approximately 50 % suggesting heterozygosity for a deletion. In patient 13, signal intensities for all 8 exons are reduced to approximately 50 % of controls suggesting heterozygosity for a deletion of the entire *FBP1* gene

Such long-range deletions and other variations in copy number, particularly when present in the heterozygous state, may escape conventional sequencing techniques. Multiplex ligation-dependent probe amplification (MLPA), originally described in 2002 [17], is increasingly used for the targeted screening for copy number variations and has recently become commercially available for the *FBP1* gene. Therefore, we applied this method to the five patients in whom we had not arrived at a diagnosis with standard sequencing techniques. Patients #1 to #3 all showed the typical pattern of homozygosity for an exon 2 deletion (Fig. 2b), thus, MLPA analysis was in accordance with our sequencing results. In patient #12, we found that MLPA for exon 8 was diminished to approximately 50 % of normal controls (Fig. 2c). Therefore, heterozygosity for a long-range deletion was supposed, which was subsequently confirmed by SNP array analysis (Table 2). In patient #13, heterozygosity for a deletion on the paternal allele was confirmed and we could show that the deletion affects all 8 exons (Fig. 2d). Furthermore, we were able to demonstrate by SNP array analysis that the mutation in pt #13 affecting the entire *FBP1* gene is not identical to the one reported by Asberg [18] who described a patient with a deletion of the entire *FBP1* gene together with the neighboring *FBP2* and *ONPEP* genes (Table 2).

## Conclusions

In summary, we provide an update of the 35 *FBP1* mutations reported to date, present PCR conditions that allow detection of a common *FBP1* mutation in the Armenian and Turkish population, and more generally, demonstrate for the first time the useful role of MLPA analysis in the diagnosis of FBP1 deficiency.

## Additional files

Additional file 1: Figure S1. Origin of the 3 patients with deletion of exon 2 of FBP1. (DOC 125 kb) Additional file 2: Figure S3. Haplotype analysis for a common long-range deletion of FBP1 in three patients from Armenia and Turkey. (DOC 50 kb)

## Abbreviations

asn: antisense
FBP1: fructose-1,6-bisphosphatase
MLPA: multiplex ligation-dependent probe amplification
PCR: polymerase chain reaction
sn: sense
