# Supplementary material for: A summary of molecular genetic findings in fructose-1,6-bisphosphatase deficiency with a focus on a common long-range deletion and the role of MLPA analysis
Source: Orphanet J Rare Dis. 2016 Apr 21;11:44. doi: 10.1186/s13023-016-0415-1 (PMC4839065; doi:10.1186/s13023-016-0415-1)
Supplement: Additional file 1: Figure S1. — Origin of the 3 patients with deletion of exon 2 of FBP1. (DOC 125 kb) [file 13023_2016_415_MOESM1_ESM.doc]

Santer *et al.*


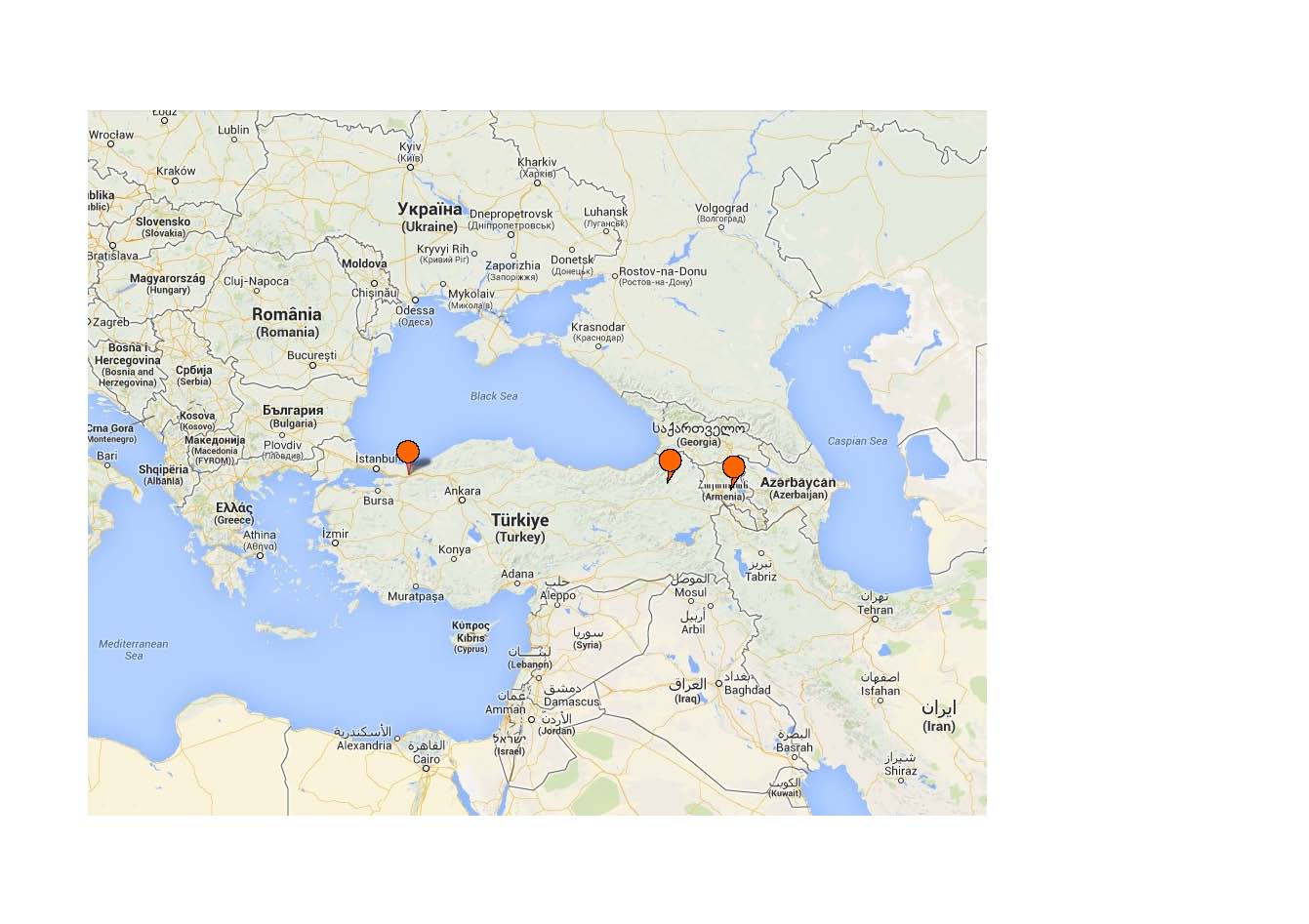


**#1**

**#2**

**#3**

A summary of molecular genetic findings in fructose-1,6-bisphos­phatase deficiency with a focus on a common long-range deletion

and the role of MLPA analysis

**Supplementary Figure 1.** Origin of the 3 patients with a deletion of exon 2 of *FBP1.*
