# Supplementary material for: A summary of molecular genetic findings in fructose-1,6-bisphosphatase deficiency with a focus on a common long-range deletion and the role of MLPA analysis
Source: Orphanet J Rare Dis. 2016 Apr 21;11:44. doi: 10.1186/s13023-016-0415-1 (PMC4839065; doi:10.1186/s13023-016-0415-1)
Supplement: Additional file 2: Figure S3. — Haplotype analysis for a common long-range deletion of FBP1 in three patients from Armenia and Turkey. (DOC 50 kb) [file 13023_2016_415_MOESM2_ESM.doc]

C

C

G

C

G

C

A

T

5’UTR (18) [rs2296707]

c.426+7 [rs8192689]

c.567+31 rs3739747]

c.651 [p.(=), rs1042144]

c.653 [p.(Arg218Lys), rs1769259]

c.705+14 [rs2297084]

c.960 [p.(=), rs1769257]

c.*213 [rs9695]

Santer *et al.*

A summary of molecular genetic findings in fructose-1,6-bisphos­phatase deficiency

with a focus on a common long-range deletion

and the role of MLPA analysis

**Supplementary Figure 2**

**Haplotype analysis for a common long-range deletion of *FBP1* in three patients from Armenia and Turkey**

Analysis of Intragenic SNPs of the *FBP1* gene in the families of patients 1 (right), 2 (middle), and 3 (left). The haplotype associated with the deletion of exon 2 is shown on the red allele.

Bold letters represent deviation from wild-type (*wt*) sequence (NM_001127628); *n.d.,* not determined

*n.d.*

**T**

G

**T**

**A**

C

**G**

**C**

*n.d.*

**T**

G

**T**

**A**

C

**G**

**C**

**T**

**T**

G

**T**

**A**

C

**G**

**C**

**T**

C

**A**

C

**A**

**T**

**G**

T

*n.d.*

**T**

G

**T**

**A**

C

**G**

**C**

*n.d.*

C

G

C

**A**

C

A

**C**

*n.d.*

**T**

G

**T**

**A**

C

**G**

**C**

*n.d.*

**T**

G

**T**

**A**

C

**G**

**C**

*n.d.*

**T**

G

**T**

**A**

C

**G**

**C**

*n.d.*

C

**A**

C

**A**

**T**

**G**

T

*n.d.*

**T**

G

**T**

**A**

C

**G**

**C**

*n.d.*

C

**A**

C

**A**

**T**

**G**

T

*n.d.*

**T**

G

**T**

**A**

C

**G**

**C**

*n.d.*

**T**

G

**T**

**A**

C

**G**

**C**

*n.d.*

**T**

*n.d.*

**T**

**A**

C

**G**

**C**

*n.d.*

C

*n.d.*

C

G

**T**

A

T

*n.d.*

**T**

*n.d.*

**T**

**A**

C

**G**

**C**

*n.d.*

C

*n.d.*

C

G

C

A

**C**

(**pt 1**, 0879-12)

(1301-13)

(1302-13)

(2428-14)

(2427-14)

(3001-14)

(3002-14)

(**pt 2**,2426-14)

(**pt 3**, 2447-14)

*wt*
